# Androgen Deprivation Therapy–Induced Cardiovascular Disease

**DOI:** 10.1200/JGO.2016.006015

**Published:** 2016-07-27

**Authors:** Manikandan Dhanushkodi

**Affiliations:** Cancer Institute (WIA), Chennai, India

## To the Editor

Essien et al^1^ reported an interesting study, “Cardiovascular disease risk factors: How relevant in African men with prostate cancer receiving androgen-deprivation therapy?” We have a few questions regarding the study.

Was there any difference in the median age between those who underwent androgen-deprivation therapy (ADT) and treatment-naïve patients? What was the gonadotropin-releasing hormone agonist used and at what interval?

Why did the patients who underwent surgical ADT have lesser incidence of cardiovascular risk factors? Was blood pressure assessed in this study, and did patients receiving ADT have higher blood pressure? Did patients receiving ADT with higher cardiovascular disease risk factors experience any cardiovascular events?

Why did the patients who underwent orchidectomy have statistically significantly higher prostate-specific antigen? It would have been informative if we had the baseline glucose, lipid profile, and indices of obesity for patients receiving ADT to show that their cardiovascular risk factors definitely increased with ADT. As there is a definite increase in cardiovascular risk factors with ADT, we should advise lifestyle modifications and/or lipid-lowering agents before initiating therapy.
